# Fern-Like Pattern on Ultra-Widefield Fluorescein Angiography Across Various Uveitic Conditions

**DOI:** 10.22336/rjo.2025.16

**Published:** 2025

**Authors:** Vipin Rana, Meenu Dangi, Pradeep Kumar, Ashish Markan, Jaya Kaushik, Ranjit Goenka, Anupama Rana, Atul Gupta, Amit Nandan Tripathi

**Affiliations:** 1 Department of Ophthalmology, Command Hospital (Eastern Command), Kolkata, India; 2 Department of Ophthalmology, Command Hospital (Central Command), Lucknow, India; 3 Department of Ophthalmology, Army Hospital Research and Referral, New Delhi, India; 4 Vitreoretina and Uvea Services, Eye Hospital, Lajpat Nagar, New Delhi, India; 5 ECHS Polyclinic, Alipore, Kolkata, India; 6 Western Eye Hospital, Marlybone Road, London, U.K.

**Keywords:** fern-like pattern, ultra-wide field fluorescein angiography, uveitis, FLP = Fern-like pattern, UWFA = Ultra-wide field Fluorescein Angiography, BD = Behçet’s disease, TB = Tubercular, VKH = Vogt-Koyanagi-Harada, BCVA = Best-corrected visual acuity, IOP = Intraocular pressure

## Abstract

**Objective:**

To investigate the occurrence and characteristics of the “Fern-like pattern” (FLP) on ultra-wide field fluorescein angiography (UWFA) across a variety of uveitic conditions, challenging the traditional association of FLP primarily with Behçet’s disease (BD).

**Methods:**

This observational study at a tertiary care centre in India analysed the UWFA images of 23 eyes from 12 patients diagnosed with BD, tubercular vasculitis, intermediate uveitis, Vogt‒Koyanagi‒Harada (VKH) syndrome, or exudative retinal detachment. Clinical and imaging data were reviewed to assess the presence and implications of FLP in these conditions.

**Results:**

The study included 12 patients: 8 males (66.67%) and four females (33.33%). The distribution was: 3 with BD, 4 with tubercular vasculitis, 3 with intermediate uveitis, 1 with VKH syndrome, and 1 with exudative retinal detachment. The average age was 29.58 ± 11.35 years. Anterior segment examination revealed significantly more hypopyon, cells, and posterior synechiae in BD patients. Posterior segment examination showed choroiditis exclusively in non-Behçet’s patients and considerably increased vitritis in BD patients. Disc hyperfluorescence and neovascularization were substantially more common in BD patients.

**Discussion:**

Our study is the first to comprehensively assess the presence of FLP on UWFA across both BD and non-BD uveitic conditions, demonstrating its occurrence in TB vasculitis, VKH syndrome, and intermediate uveitis. While traditionally linked to BD, FLP signifies retinal capillary leakage and inflammation, necessitating careful differential diagnosis. We observed optic disc hyperfluorescence predominantly in BD, underscoring its role as a potential disease activity marker requiring aggressive immunosuppression. Notably, FLP was identified in exudative retinal detachment and VKH, suggesting shared inflammatory mechanisms across conditions. These findings broaden the clinical relevance of FLP in uveitis and highlight the need for further studies to explore its diagnostic and prognostic significance.

**Conclusion:**

The study confirms that FLP on UWFA is not exclusive to BD and appears in other uveitic conditions. Clinical signs such as hypopyon and disc hyperfluorescence indicate BD, whereas choroiditis is more common in non-Behçet’s uveitis. These findings highlight the importance of precise clinical and imaging assessments to distinguish between different uveitic conditions for accurate diagnosis and effective treatment.

## Introduction

The “Fern-like pattern” (FLP) on fluorescein angiography (FA) is a notable imaging finding characterized by diffuse retinal capillary leakage [1]. Traditionally, FLP has been closely associated with Behcet’s disease (BD), manifesting during acute episodes of Ocular Behcet’s disease or indicating an inadequate response to therapy in clinically quiescent phases [2,3]. Its identification in BD patients is a crucial diagnostic marker, guiding therapeutic strategies and interventions.

Despite its established connection to BD, recent observations have also documented the occurrence of FLP in a range of non-Behcet’s disease conditions, such as tubercular (TB) vasculitis and intermediate uveitis. This broadening of associated conditions challenges the traditional diagnostic view that primarily links FLP to BD and suggests more extensive clinical significance. There is a noticeable lack of detailed literature exploring FLP characteristics across different uveitic entities.

This study aims to fill this gap by systematically describing the clinical and imaging characteristics of FLP on ultra-wide-field fluorescein angiography (UWFA) across various uveitic conditions, including Behcet’s disease, TB vasculitis, intermediate uveitis, and Vogt-Koyanagi-Harada (VKH) syndrome. By doing so, we hope to enhance the diagnostic accuracy and management of retinal vasculitis and related conditions.

## Materials and methods

This observational study was performed at the Retina and Uveitis Services of a tertiary care center in India. It involved a retrospective review of the clinical records of patients demonstrating FLP of capillary leakage on UWFA. The study adhered to the tenets of the Declaration of Helsinki and was approved by the institutional Ethics Committee.

### Study Subjects

Subjects with FLP on UWFA between July 2022 and April 2024 were enrolled in the study.

### Study Procedures

The medical records of all the study subjects were reviewed. The detailed clinical history, which included the presence of oral ulcers, genital ulcers, skin lesions, a history of TB or any TB contact, and complete ophthalmological evaluation comprising best-corrected visual acuity (BCVA) assessment by Log MAR, intraocular pressure (IOP) measurement, slit-lamp biomicroscopy evaluation, and relevant laboratory investigations, was reviewed. The anterior segment findings studied included the presence/absence of cells, flares, granulomatous or nongranulomatous keratic precipitates, posterior synechiae, and iris nodules. The posterior segment findings studied were the presence/absence of vitreous cells, vitreous haze, snowballs, vascular sheathing or perivascular exudates, retinitis, choroiditis, and disc edema. Fundus imaging with UWF color fundus photography, UWFA (Optos California ultrawide field imaging 200 DTx icg, Scotland, United Kingdom), and swept source-optical coherence tomography (DRI Triton® Topcon, Topcon Inc., Japan) were analyzed for all the subjects. On UWFA, the presence of FLP and simultaneous involvement of an artery, vein, or both; location of vascular leakage (posterior pole, periphery, or both); disc hyperfluorescence; retinitis; choroiditis; neovascularization elsewhere; and neovascularization of the optic disc were noted. All eyes without FLP on UWFA were excluded from the study.

The diagnosis of BD was made using the currently used classification, which requires the presence of recurrent oral ulcers plus any two of the following: skin lesions, recurrent genital ulcers, ocular manifestations, or a positive pathergy test [4]. The diagnosis of TB vasculitis was made according to the COTS study [5].

### Statistical analysis

Statistical analysis was performed using the Statistical Package for Social Sciences (SPSS version 20 for Windows). Descriptive statistics were calculated for continuous variables, including the means and standard deviations. Frequencies and percentages were computed for categorical data such as clinical signs and symptoms (e.g., hypopyon, keratic precipitates). The associations between categorical variables across different groups (Behçet’s disease versus non-Behçet’s uveitic conditions) were evaluated using the chi-square or Fisher’s exact test where applicable. For continuous variables, comparisons between two independent groups were made using the Mann-Whitney U test due to the nonparametric nature of the data distribution. A p-value of < 0.05 was considered significant for all the tests.

## Results

### Baseline Demographics of the Patients

**Fig. 1:**
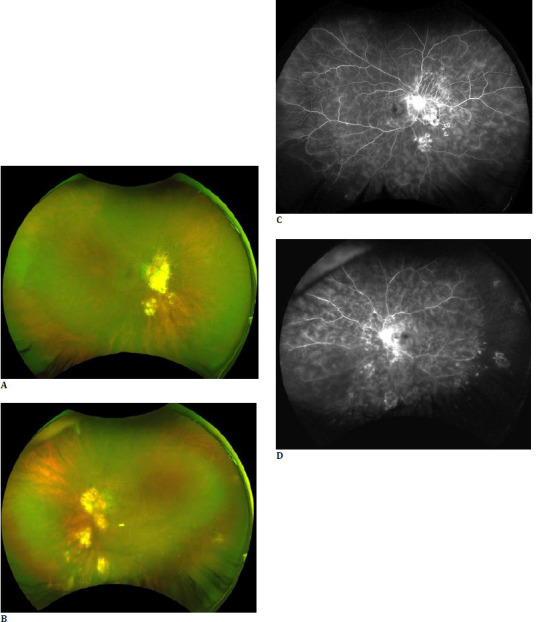
45-year-old female diagnosed with tubercular retinal vasculitis in both eyes, displaying vitritis and peripapillary and perivascular choroiditis scars in both eyes (**A, B**). Fundus Fluorescein angiography of both eyes reveals staining of the choroiditis scars, capillary leakage in a fern-like pattern, and areas of capillary non-perfusion in the periphery of the left eye (**C, D**).

**Fig. 2:**
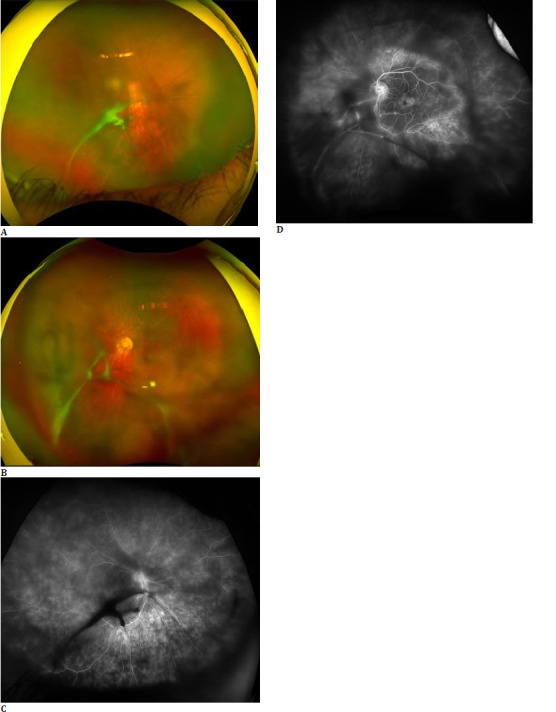
16-year-old boy diagnosed with intermediate uveitis in both eyes, demonstrating dense vitritis in both eyes (**A, B**). Fundus Fluorescein angiography of both eyes reveals a fern-like pattern and a flower petal appearance in the fovea, indicative of cystoid macular edema (**C, D**).

**Fig. 3:**
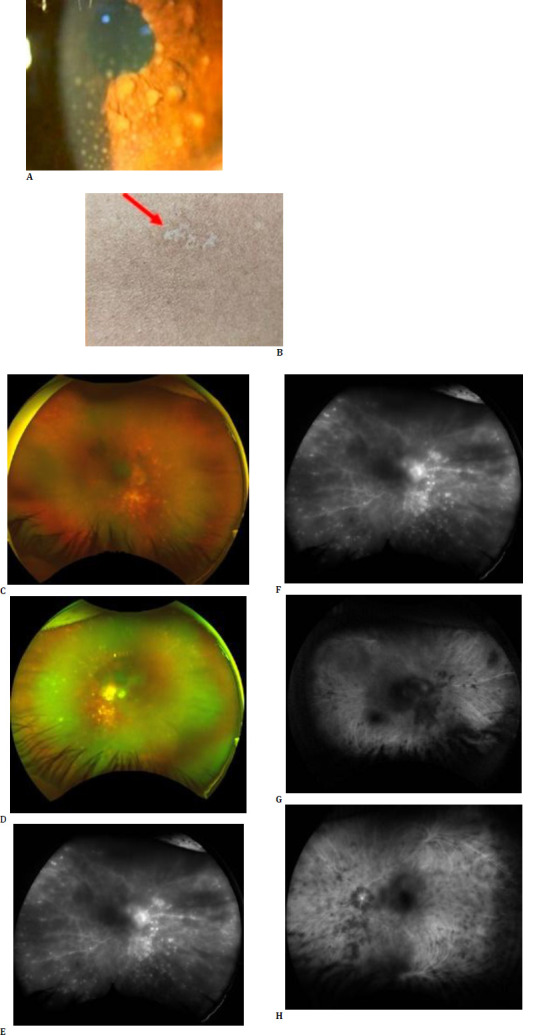
38-year-old female patient with a history of Vogt-Koyanagi-Harada (VKH) syndrome, currently treated with Azathioprine, presenting with a recurrence. The clinical findings include granulomatous keratic precipitates and Busacca nodules (**A**), vitiligo (**B**), and vitritis in both eyes (**C, D**). Fundus Fluorescein angiography reveals a fern-like pattern in both eyes (**E, F**). Indocyanine green angiography shows hypofluorescent spots, which are more pronounced in the late phase and indicative of full-thickness choroidal granulomas (**G, H**).

**Fig. 4:**
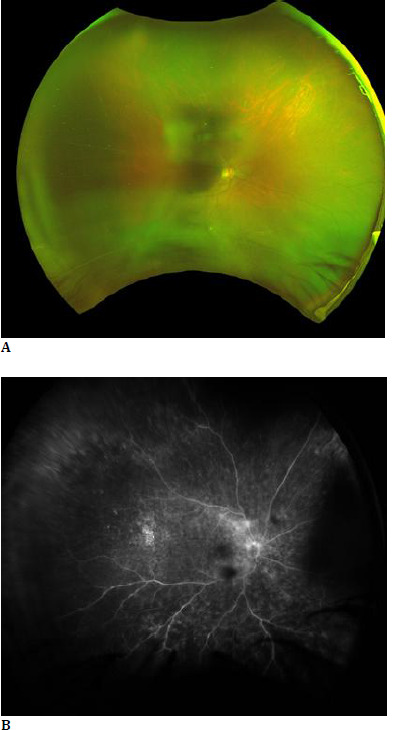
50-year-old male patient diagnosed with exudative retinal detachment, presenting with vitritis in the right eye (**A**). Fundus Fluorescein angiography of the right eye shows an inferior fern-like pattern, corresponding to the area of exudative detachment (**B**).

We analysed the eyes of 23 patients, including eight males (66.67%) and four females (33.33%). The patients were distributed as follows: 3 with BD, 4 with TB vasculitis (**Fig. 1 A-D**), 3 with intermediate uveitis (**Fig. 2 A-D**), 1 with VKH syndrome (**Fig. 3 A-H**), and 1 with exudative retinal detachment (**Fig. 4 A, B**). The average age was 29.58 ± 11.35 years, with 11 patients (91.67%) showing bilateral involvement and one patient (8.33%) showing unilateral involvement.

### Clinical History and Systemic Findings

Oral ulcers and genital ulcers were exclusively reported in all patients with BD. A history of TB or TB contact was observed in all patients with TB vasculitis. VKH patients presented with sensorineural loss and vitiligo patches (**Fig. 3 B**). There were no significant systemic findings in patients with intermediate uveitis or exudative retinal detachment.

### Anterior Segment Findings (**Table 1**)

Keratic precipitates were present in 66.67% of BD patients and 47.06% of non-Behcet’s patients (P=0.640). Cells were significantly greater in BD patients (100%) than in non-Behcet’s patients (47.06%) (P=0.048). Posterior synechiae were more common in BD patients (66.67%) compared to non-Behcet’s patients (11.76%) (P=0.021). Hypopyon was found exclusively in BD patients (33.33%, P=0.059).

**Table 1:** Clinical features of Behcet’s versus non-Behcet’s group

Features	Behcet’s Disease (n=6)	%	Non-Behcet’s Group (n=17)	%	P-value
**Anterior Segment Findings**					
KP	4	66.67%	8	47.06%	0.640
Cells	6	100.00%	8	47.06%	0.048*
PS	4	66.67%	2	11.76%	0.021*
BB	0	0.00%	6	35.29%	0.144
Hypopyon	2	33.33%	0	0.00%	0.059
**Posterior Segment Findings**					
Vitritis	6	100.00%	9	52.94%	0.058
Snow banking	0	0.00%	2	11.76%	0.999
ERM	0	0.00%	2	11.76%	0.999
Retinitis	0	0.00%	0	0.00%	-
Choroiditis	0	0.00%	8	47.06%	0.058
Sheathing	2	33.33%	4	23.53%	0.632
CME	2	33.33%	4	23.53%	0.632
NVE	4	66.67%	8	47.06%	0.640
NVD	2	33.33%	0	0.00%	0.059
disc edema	0	0.00%	2	11.76%	0.999
Choroidal Neovascularisation (CNV)	0	0.00%	0	0.00%	-
Hemorrhage (HGE)	0	0.00%	0	0.00%	-

**Abbreviations**: KP = keratic precipitates, PS = posterior synechiae, BB = broad based posterior synechiae, ERM = epiretinal membrane, CME = cystoid macular edema, NVE = neovascularisation elsewhere, NVD = neovascularisation of optic disc, CNV = choroidal neovascularisation, HGE = hemorrrhage

* Significant (p-value < 0.05) Fisher’s exact test used

### Posterior Segment Findings (**Table 2**)

All BD patients had vitritis, while 52.94% of non-Behcet’s disease patients had vitritis (P=0.058). Choroiditis was absent in BD patients but was present in 47.06% of non-Behcet’s patients (P=0.058). Cystoid macular edema and neovascularization were more frequent in BD patients.

**Table 2:** Ultra-wide fundus fluorescein angiography findings in Behcet’s versus non-Behcet’s group

Feature	Behcet’s Disease (n=6)	%	Non-Behcet’s Group (n=17)	%	P-value
CNP	4	66.67%	8	47.06%	0.640
CME	2	33.33%	4	23.53%	0.632
Disc hyperfluorescence	4	66.67%	0	0.00%	0.002*
NVE	4	66.67%	8	47.06%	0.640
NVD	2	33.33%	0	0.00%	0.059
Retinitis	0	0.00%	0	0.00%	-
choroiditis	0	0.00%	8	47.06%	0.058

**Abbreviations:** CNP = capillary non-perfusion, CME = cystoid macular edema, NVE = neovascularisation elsewhere, NVD = neovascularisation of the optic disc

* Significant (p-value < 0.05) Fisher’s exact test used

### Ultrawidefield Fluorescein Angiography Findings

Disc hyperfluorescence was significantly more common in BD patients (66.67%) than in non-Behcet’s patients (P=0.002). Capillary nonperfusion and neovascularization were observed across both groups, with no significant differences (P=0.640). Neovascularization of the optic disc was found in 33.33% of BD patients but was absent in non-Behcet’s disease patients (P=0.059).

## Discussion

Our study is the first to systematically investigate FLP on UWFA in BD patients versus non-BD patients. We found that FLP, traditionally associated with BD, also occurs in non-Behcet’s uveitic conditions such as TB vasculitis, VKH syndrome, and intermediate uveitis. This broadens the clinical significance of FLP and highlights the need for careful differential diagnosis in uveitis patients.

FLP is suggestive of retinal capillary leakage and is prominent in the arteriovenous phase of FA. It helps assess the status of retinal inflammation [3]. Ramtohul et al. [6] analyzed the spectrum of perivenular fern-like leakage on UWFA in pars planitis and central retinal vein occlusion patients. They demonstrated that the anatomical origin of the perivenular FLP corresponds to the deep capillary plexus.

Ocular involvement in BD is characterized by bilateral, recurrent, non-granulomatous panuveitis and retinal vasculitis. Retinal vasculitis may involve arteries, veins, or both [2,3,5]. However, diffuse retinal capillary leakage in an FLP is the most typical FA finding, which is considered evidence of inadequate therapeutic response during clinically quiescent periods and, thus, is the gold standard for detecting and monitoring both leaky and occlusive retinal vasculitis in BD patients [3,7,8]. Other FFA findings described in BD include staining of vessel walls, diffuse leakage from small and large retinal vessels, and capillary nonperfusion areas [9,10]. We observed all these findings in our study. We observed FLP in all patients’ active disease in both the posterior pole and periphery. However, only peripheral retinal capillary leakage may be detected in asymptomatic patients without clinical activity [10].

Hyperfluorescence of the optic disc was an important finding in BD patients in our study. It has also been suggested to be a marker of disease activity and has been described in BD and non-Behcets’ disease. Mesquida et al. [11] studied thirty-eight eyes of 20 patients with BD with retinal vasculitis and reported optic disc hyperfluorescence in 62.2%. Keorochana et al. [12] also reported optic disc leakage/staining in 74% of eyes with BD. Bansal et al. [13] described subtle disc hyperfluorescence in patients with tubercular-associated uveitis. In our study, we detected disc hyperfluorescence only in BD patients. We speculate that the increased incidence of optic disc hyperfluorescence in BD patients is probably due to the increased severity of the disc. Thus, aggressive treatment with double immunosuppression/biologics is needed.

White dots or superficial white patches are foci of retinitis, retinal exudates, or retinal infiltrates [14]. Hussein et al. [15] stated that such lesions may be solitary or multifocal and variable in depth, size, and distribution within the fundus during active inflammation in BD patients. However, we did not find retinitis lesions in any of our patients.

TB retinal vasculitis is characterized by an occlusive phenotype with predominant retinal vein involvement [16]. It is characterized by perivascular sheathing with exudates and retinal hemorrhages. The presence of perivascular choroidal pigment or small choroiditis patches highly suggest TB etiology [16]. To our knowledge, the presence of these choroiditis scars, together with FLP, has rarely been studied. In our study, four patients with TB vasculitis had choroiditis scars coupled with FLP and positive radiological or immunological tests for TB.

Interestingly, our study also revealed the presence of FLP under conditions such as exudative retinal detachment and VKH syndrome, where this pattern has not been previously described in the literature. The appearance of FLP under these conditions may result from similar pathophysiological mechanisms of capillary leakage and inflammation observed in BD but manifest under different clinical scenarios. In exudative retinal detachment, fluid accumulation beneath the retina could lead to changes in vascular permeability or mechanical stress on capillaries, thereby causing a leakage pattern resembling FLP. Similarly, VKH syndrome, known for its autoimmune inflammation primarily affecting the choroid and optic nerve, might induce secondary retinal capillary leakage in a pattern miming FLP due to the extensive inflammatory response and its effect on the retinal vascular bed. Our patient was a follow-up case of recurrent VKH syndrome where typical exudative detachment was not seen, and anterior segment findings were more than the posterior segment. Further research is needed to explore these associations and understand the underlying mechanisms contributing to the manifestations of FLP under these diverse conditions.

Our study showed that FLP can also be observed in other inflammatory conditions. We speculated that the pathophysiological mechanisms underlying FLP in different uveitic conditions might involve similar inflammatory processes affecting the retinal vasculature, leading to capillary leakage.

The current study had a few limitations. First, selection bias may have occurred because this was a single-center study conducted at a tertiary care institute. Second, we had a small sample size; however, this may be explained by the rarity of FLP.

## Conclusion

In conclusion, this study confirms that FLP on UWFA extends beyond BD to include other uveitis conditions, such as TB vasculitis, intermediate uveitis, and VKH syndrome. Clinical signs like hypopyon and disc hyperfluorescence favor BD, while choroiditis is more common in non-Behcet’s uveitis. These distinctions are crucial for accurate diagnosis and effective treatment. Further research is needed to explore the mechanisms underlying FLP in various uveitic conditions.
